# Investigating Awareness and Acceptance of Digital Phenotyping in Dhaka’s Korail Slum: Qualitative Study

**DOI:** 10.2196/65530

**Published:** 2025-06-23

**Authors:** Nadia Alam, Domenico Giacco, Bulbul Siddiqi, Swaran P Singh, Sagar Jilka

**Affiliations:** 1 Warwick Medical School, The University of Warwick, Gibbet Hill Road, Coventry, CV4 7AL, United Kingdom, 44 7300311294; 2 Department of Political Science and Sociology, North South University, Dhaka, Bangladesh

**Keywords:** digital phenotyping, mental health, slums, serious mental disorders, lower- and middle-income countries, LMIC, mobile phone

## Abstract

**Background:**

Digital phenotyping (DP), the process of using data from digital devices, such as smartphones and wearable technology to understand and monitor people’s behavior, health, and daily activities, has shown significant promise in mental health care within high-income countries. However, its application in low- and middle-income countries (LMICs) is limited, particularly among impoverished populations such as slum residents.

**Objectives:**

This study investigates the awareness, knowledge, acceptance, and implementation of DP, including willingness to share data, and concerns regarding privacy and data security, among residents of Dhaka’s Korail slum, one of Bangladesh’s largest and most densely populated informal settlements. Understanding awareness, acceptance, and privacy concerns surrounding DP in these settings is critical for its effective implementation.

**Methods:**

We conducted 8 focus group discussions with 79% (30/38) of female participants (mean age 37, SD 13.7 years). Participants included 20 individuals diagnosed with serious mental disorders and 18 caregivers. The focus group discussions also included a section explaining what DP is.

**Results:**

Smartphone ownership was reported by 45% (17/38) of the participants, while 92% (35/38) of the participants had access to a smartphone through family members. There was a general lack of awareness about DP among the participants. Initially, 92% (35/38) of participants had no prior knowledge of DP, but after receiving an explanation, they acknowledged its potential applications and benefits. Participants recognized the use of DP for health monitoring, particularly in managing mental health conditions. Participants expressed willingness to share certain types of data, particularly phone usage and location data, provided that content-level information remained private. Despite these perceived benefits, significant concerns about privacy and data security emerged. Participants expressed fears about the potential misuse of their personal information, with some feeling resigned to the idea of already being constantly monitored. Trust in DP tools emerged as a critical factor for adoption, highlighting the need for transparent data protection policies and user control over data sharing. Additionally, participants emphasized the importance of adapting DP tools to local contexts, including cultural considerations and technological literacy.

**Conclusions:**

While DP presents a promising avenue for mental health support in underserved urban populations, its adoption in LMIC slum settings requires targeted educational initiatives, robust privacy safeguards, and community involvement to ensure trust and usability. DP tools should be adapted to fit the cultural context of the target population, possibly involving modifications to the types of data collected or the way data are interpreted. While DP holds potential to improve mental health care in underserved communities, addressing barriers related to awareness, privacy, culture, and usability is crucial. Focusing on educational initiatives, robust data protection, cultural adaptation, user-friendly design, and community engagement, DP can become a valuable tool in bridging the mental health care gap in LMICs.

## Introduction

### Background

Mental health disorders constitute a substantial global health challenge, with approximately 80% of individuals affected residing in low- and middle-income countries (LMICs) [1]. Factors such as rapid urbanization, poverty, and frequent exposure to traumatic events contribute to the high prevalence of mental health problems in these settings. For instance, in the Korail slum of Dhaka, Bangladesh, the combination of poor living conditions, high population density, and limited access to mental health services exacerbates the prevalence and impact of serious mental disorders (SMDs) such as schizophrenia and major depressive disorder [2-4].

The mental health care gap in LMICs is stark. In Bangladesh, for example, the mental health workforce is critically insufficient, with fewer than 0.5 psychiatrists per 100,000 people [5]. Consequently, many individuals with mental health conditions remain untreated, leading to significant personal and societal costs [1]. This treatment gap is particularly pronounced in urban slums where health care infrastructure is weakest and social determinants of health are most severe.

Korail is one of the largest slums in Dhaka, Bangladesh, with an estimated population of around 200,000 people [6]. It is located in the heart of the city, near affluent neighborhoods and commercial hubs, making it a focal point for discussions about urban inequality [6,7]. The slum is marked by overcrowded and informal housing structures, most of which are constructed from makeshift materials such as bamboo, plastic sheets, and tin [6]. These homes lack basic amenities such as proper sanitation, clean water, and stable electricity, which exacerbates the residents’ vulnerability to public health crises and environmental hazards [8].

The population of Korail is highly diverse, consisting largely of internal migrants from rural areas who come to Dhaka in search of employment [8,9]. Many work informally as domestic laborers or small-scale street vendors [6,9]. The socioeconomic diversity within Korail still reflects varying degrees of poverty, with some households relatively better off due to more stable employment or business opportunities, while others remain in extreme deprivation [2,6].

The living conditions in Korail pose significant barriers to health care access, including mental health services [6,8]. The lack of formal health care infrastructure means that residents often rely on informal providers, traditional healers, or nongovernmental organizations for medical help [4]. This makes the introduction of innovative health interventions such as digital phenotyping (DP) both a potential opportunity and a challenge. Given these challenges, implementing digital health solutions requires careful consideration of these unique socioeconomic and cultural factors [3,4].

Korail serves as a microcosm of the broader challenges facing slum communities across LMICs, such as rapid urbanization, extreme poverty, and limited access to essential services [9]. This setting provides a critical backdrop for examining the potential of DP in LMICs, while also highlighting the specific barriers that need to be addressed to make such interventions feasible and effective.

Digital mental health tools could provide a solution, and DP could revolutionize mental health care in LMICs by providing low-cost, scalable methods for early detection, monitoring, and intervention of mental health disorders [10]. The widespread use of smartphones even in impoverished areas offers a unique opportunity to collect valuable data on individuals’ behaviors and environments. These data can be used to identify early warning signs of relapse in SMDs, track treatment progress, and personalize interventions [10].

Studies in high-income countries (HICs) have demonstrated the efficacy of DP in various mental health contexts [11]. For instance, passive data from smartphones, such as GPS and call logs, have been used to predict relapse in schizophrenia [12]. These methodologies, however, need to be adapted for LMIC settings, where cultural norms, technological access, and health care infrastructure differ markedly. For instance, communication patterns, social interactions, and even the use of technology can differ widely between populations in HICs and those in LMICs [3]. There is often a lack of understanding and awareness about digital health technologies among the general population and health care providers in LMICs [3]. In the Korail slum, while smartphone usage is common, the concept of DP might be largely unknown. This gap in knowledge necessitates educational initiatives to inform communities about the benefits and implications of DP. These initiatives should aim to demystify the technology, explaining how it works and how it can be used to improve mental health outcomes.

### Importance of Context-Specific Research

Understanding the level of awareness, knowledge, and acceptance of DP among residents of LMICs is essential for the successful implementation of digital health interventions [13]. Awareness and acceptance are crucial as limited understanding can hinder adoption and effectiveness [1], with skepticism about sharing personal data posing a significant challenge [14]. Cultural attitudes toward mental health and technology also play a significant role, as stigmatization and cultural beliefs can impact perceptions of DP [15]. Addressing data privacy concerns is critical, as transparency and robust protection measures can enhance trust [16,17]. Engaging the community in the design and implementation of DP tools through participatory approaches can improve usability and relevance, ensuring that interventions meet the specific needs and preferences of the population [18], thereby increasing their sustainability.

While DP has shown promise in HICs, its adoption in LMICs remains limited, and no qualitative research exists [19] to understand people’s perceptions and acceptance of this technology in these settings. Understanding these perceptions is vital for developing culturally sensitive and effective DP that can improve mental health outcomes in these underserved populations.

There is currently limited research on the awareness, knowledge, and acceptance of DP in LMICs, particularly among slum populations. To effectively implement DP in LMIC settings, it is crucial to first understand the perspectives of the target population. This study seeks to address this gap by exploring the perceptions of DP among residents of Dhaka’s Korail slum. By identifying the key barriers to adoption and the factors that influence acceptance, this study will contribute to bridging the mental health care gap in underserved populations. Through focus group discussions (FGDs) with individuals diagnosed with SMDs and their caregivers, this research aims to provide crucial insights into the potential of DP as a tool for mental health care in resource-poor settings. The findings will inform the development of culturally sensitive, secure, and user-friendly DP tools adapted to meet the specific needs of slum communities in LMICs. This study aims to explore the awareness, knowledge, and acceptance of DP among residents of the Korail slum in Dhaka, Bangladesh, through FGDs.

## Methods

### Study Design

This study uses a qualitative research design to explore the awareness, knowledge, and acceptance of DP among residents of the Korail slum in Dhaka, Bangladesh. The qualitative approach was chosen to gather in-depth insights into the participants’ perceptions and attitudes toward DP. This study followed the COREQ (Consolidated Criteria for Reporting Qualitative Research) checklist to ensure comprehensive and transparent reporting of the qualitative research process [20]. This study is part of the larger National Institute for Health and Care Research’s “Transforming access to care for serious mental disorders in slums” project (TRANSFORM), which outlines the overall workflow [4]. The aim of the TRANSFORM project is to improve access to care for individuals with SMDs living in urban slum settings [4]. Specifically, the study focuses on addressing the treatment gap by implementing and evaluating innovative, community-based interventions. The project seeks to generate evidence on effective, scalable approaches to delivering mental health care in resource-limited urban slum contexts, ultimately aiming to enhance mental health outcomes for underserved populations [4].

### Study Setting and Recruitment

Participants were recruited from the Korail slum, one of the largest and most densely populated slums in Dhaka. Participants were selected using purposive sampling. Participants were identified through the TRANSFORM project where community engagement representatives are well known and respected in the area. Given the lack of formal mental health services in the area, recruitment strategies were carefully designed to ensure ethical engagement and inclusivity. A community-based approach was used to identify and enroll participants, leveraging existing partnerships with local stakeholders. We collaborated with community health workers, nongovernmental organizations, and TRANSFORM [4] community representatives, who had well-established relationships with the residents. These trusted individuals facilitated introductions and provided culturally appropriate explanations of the study to potential participants. In addition, community awareness sessions were organized in local field offices, tea stalls, and women’s group meetings, where discussions focused on mental health challenges and introduced the concept of DP in an accessible way. The consent process was structured to ensure informed decision-making. Potential participants were provided with a detailed study information sheet in both English and Bengali, outlining the study objectives, procedures, and risks. Participants were given at least 24 hours to consider their involvement before providing written informed consent. For those with limited literacy, the consent form was read aloud, and thumbprint consent was obtained in the presence of a witness. The inclusion criteria were residents of the Korail slum, aged 18 years or older, and either diagnosed with an SMD or caregivers of individuals diagnosed with SMD.

### Ethical Considerations

#### Human Participant Ethics Review Approvals

This study was reviewed and approved by the biomedical and scientific research ethics committee at the University of Warwick (reference: BSREC 100/22‐23). As part of the broader National Institute for Health and Care Research–funded TRANSFORM project [4], the study protocol underwent rigorous ethical scrutiny, including local oversight by institutional partners in Bangladesh, to ensure compliance with country-specific research ethics requirements.

We developed a safeguarding and risk policy to ensure the safety and well-being of participants during their participation in this study. This research involved discussion and questions on mental illness and individual’s own or a family member’s experience. Such discussions may include distressing events and experiences, which may cause emotional distress. Data collectors NA (a female doctoral candidate from Dhaka based in England) and SR (a male qualitative researcher from Dhaka) were trained in qualitative data collection and had a background in psychology with training in research ethics and conducting research with sensitive population. Our safeguarding policy included actions to respond to events if a participant was uncomfortable or vocalized their distress. This included pausing or stopping data collection, checking in with the participant, and in cases where a participant required professional assistance, the study team, which included local psychiatrists, was available to provide additional support.

#### Informed Consent

Written informed consent was obtained from all study participants prior to data collection. Participant information sheets and consent forms were provided in both English and Bengali, ensuring clarity and accessibility for the target population. Potential participants were given at least 24 hours to review study materials before consenting. For individuals with lived experience of mental illness, a capacity assessment tool, previously validated in the TRANSFORM study [4], was used to ensure their ability to provide informed consent. In cases where participants were unable to sign, a thumbprint was collected with a witness present. Verbal consent was also recorded at the start of FGDs. Participants were informed that they could withdraw their data without providing a reason.

#### Privacy and Confidentiality

All data collected during this study were anonymized and stored on encrypted devices in compliance with the General Data Protection Regulation and the UK Data Protection Act (2018). Identifiable data, such as signed consent forms, were securely stored in locked cabinets separate from research data. Transcripts of FGDs were deidentified before analysis, and no personally identifiable information was included in study outputs. All digital data were stored on a secure university server with restricted access limited to the research team.

#### Compensation

To acknowledge participants’ time and effort, a remuneration of 800 Bangladeshi taka (~US $6.60 or £5.30) was provided for each participant. This compensation was determined based on local standards and covered potential costs such as transportation and time off work. The Korail slum is large urban area, which requires lengthy travel, so participants were also remunerated for their cost of local transport (rickshaw) to the field office. Participants were explicitly informed that compensation was not contingent upon completing the study and could be retained even if they chose to withdraw.

#### Participant Identification in Study Materials

No identifiable personal data of individual participants will be published in the manuscript or supplementary materials. All study data were anonymized before analysis, and participant identifiers were replaced with unique codes.

### Procedure

#### Topic Guide Development

Prior to facilitating FGDs, we codeveloped the interview topic guide in collaboration with members of the Korail community, people with lived experience, and their family members. Community members were engaged through structured discussions to ensure that their perspectives were meaningfully integrated. We discussed the topic of digital mental health and through this discussion, we generated an outline to explain the concept of DP. Alongside this, we also reviewed the literature on DP in LMICs to identify existing themes and gaps in the knowledge base. The initial draft of the topic guide was iteratively refined through feedback sessions with the community, ensuring that it aligned with their priorities and concerns. Where necessary, adjustments were made to improve clarity and relevance. The final guide was designed and generated to facilitate open-ended, meaningful discussions on digital mental health and DP, reflecting both academic insights and lived experiences.

#### Focus Group Discussions

The FGDs were conducted in the field office of the TRANSFORM Project. The FGDs were structured to ensure a diverse representation of the community, including different genders, ages, and socioeconomic status. Each session was moderated by a trained facilitator, with the assistance of a notetaker. The moderator guided the discussions using a semistructured topic guide, which included questions about participants’ understanding of DPs, their current use of digital devices, their willingness to share data, and their concerns regarding privacy and data security (see supplementary information for topic guide in Multimedia Appendix 1), and lasted for a duration of 30‐45 minutes. Participants were asked about their understanding and views on DP, including its perceived usefulness, comfort levels with data sharing, potential community acceptance, and DP-related concerns.

To ensure that all participants had a similar baseline knowledge of the DP, prior to the start of the FGD, DP was explained in simple Bengali language as follows:

*Digital phenotyping is a method through which data is collected from smartphones and other smart devices to understand individual’s behavior from their regular daily activities. Researchers can use this data to study social cohesion and interactions, behavioral patterns, speech, mobility etc., which are known as digital phenotypes*.

*Data from the phone includes phone usage, location, use of social media (such as Facebook, TikTok). An application is installed on participants’ phones after explaining what sort of data will be collected from them, which will allow researchers to collect that data. The data will only be accessible to those involved in the study. The data generated by the app can be used to identify potential mental health problems which can be understood through phone usage patterns*.

#### Data Collection

The FGDs were audio recorded to ensure accurate capture of the discussions. The recordings were then transcribed verbatim and translated into English. To maintain confidentiality, all personal identifiers were removed during transcription. The translated transcripts were reviewed by the research team to ensure accuracy and fidelity to the original conversations.

### Data Analysis

**Figure 1.**
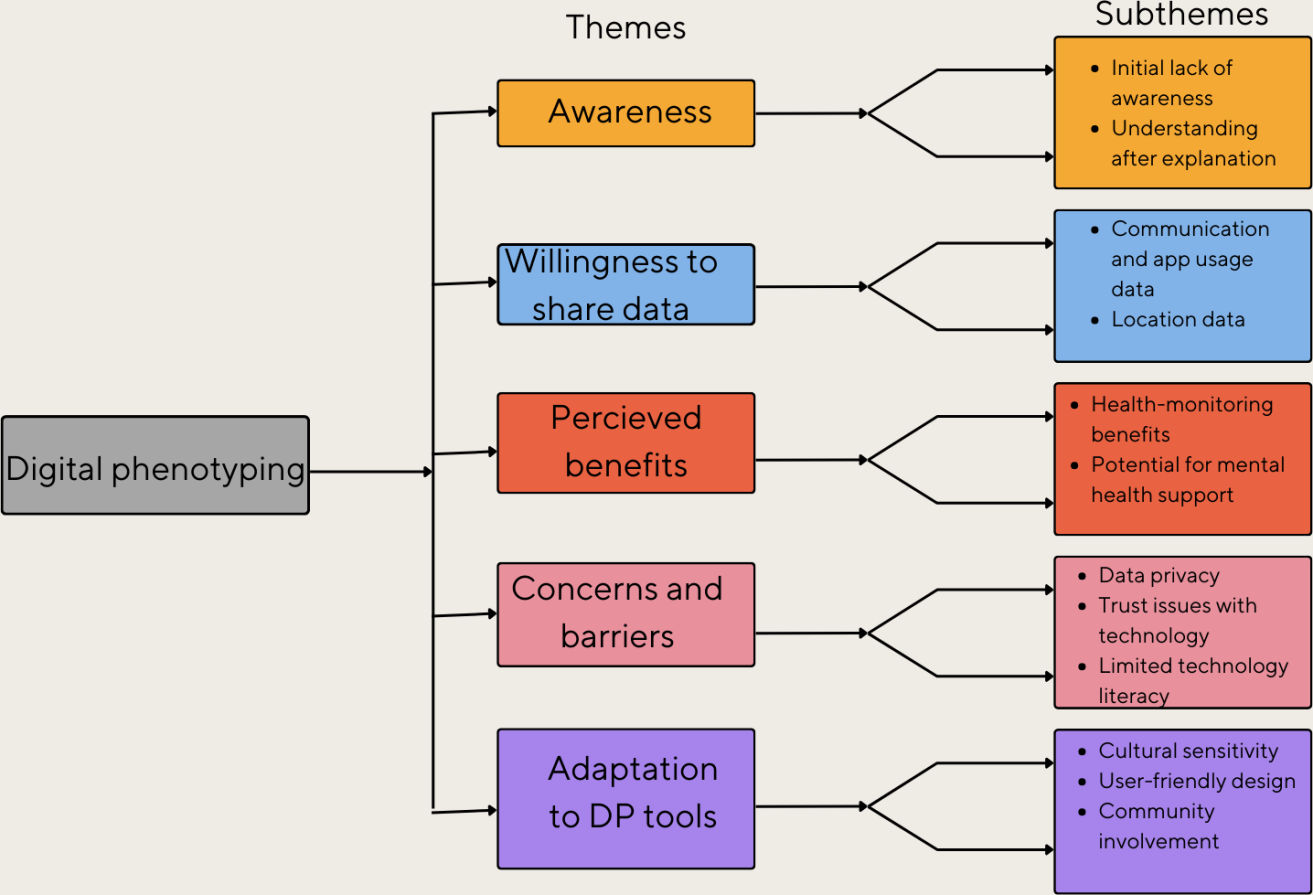
Coding tree illustrating key themes and subthemes derived from the thematic analysis of focus group discussions on digital phenotyping awareness and acceptance among individuals with serious mental disorders and their caregivers in the Korail slum, Dhaka, Bangladesh. DP: Digital phenotyping.

The qualitative data from the FGDs were analyzed using NVivo 14 software. A thematic analysis approach was used to identify patterns and themes within the data. The analysis followed the 5 stages of data analysis [21]: familiarization, identifying a thematic framework, indexing, charting, and mapping and interpretation. Each transcript was read multiple times to enhance familiarity with the data. Initial thoughts, reflections, and preliminary codes were noted. The transcript was then read again and preliminary themes were recorded. Preliminary themes were subsequently grouped into clusters based on common features and meanings. A coding tree was developed to systematically organize these themes, illustrating the hierarchical relationships between overarching themes, subthemes, and specific codes. This structure allowed for a more nuanced understanding of participant experiences and was iteratively refined by the research team. The final coding tree is shown in Figure 1. These themes were validated by cross-checking with the transcript to ensure that they accurately represented the data. This process was repeated for each transcript to maintain consistency. The themes from all transcripts were then compared and combined into master themes to create a comprehensive portrayal of the participants’ experiences. The master themes were checked and rechecked against the transcripts to ensure that they were well represented and grounded in the data. Commonalities among the preliminary themes were identified and represented as subthemes, reflecting lower-order aspects of the master themes. To enhance confirmability [22], the findings were grounded in the data by using verbatim quotes from participants to support the identified themes. Dependability [22] was ensured by maintaining a detailed audit trail, including records of coding decisions and analytical memos. The final coding framework was reviewed and refined by 3 researchers (NA, SR, and SJ) to ensure reliability and validity. Discrepancies were resolved through discussion and consensus.

Reflexivity [23] was a critical component of the analysis process. The researchers’ positionalities, including their professional backgrounds and cultural contexts, were acknowledged throughout the study to minimize bias. Regular team discussions were held to reflect on the potential influence of these factors on data interpretation. This reflexive practice ensured that the analysis remained grounded in the participants’ narratives. Furthermore, credibility [22] was enhanced through triangulation and peer debriefing. The researchers regularly discussed emerging themes and cross-checked identified themes.

Transferability [22] of the study’s findings was facilitated through detailed descriptions of the research setting and participant demographics. The narratives from Dhaka’s Korail slum enable to assess the applicability of the findings to similar urban slum environments or other resource-limited and impoverished settings. Additionally, the transparency of the thematic analysis process, coupled with the use of participant quotes to support identified themes, strengthens the study’s relevance for broader application in comparable contexts. While the findings are rooted in Korail, the themes identified may offer valuable insights for impoverished communities across LMICs.

## Results

### Demographic Information

Eight FGDs were held with homogenous groups of either people with lived experiences or their caregivers and each FGD included 4‐6 participants. There was a total of 38 participants, with an average age of 37 years (SD 13.7). All participants invited to participate took part in the study and no repeat interviews were carried out. Participants were predominantly women (30/38, 79%) and educated to primary (11/38, 29%) or secondary level (16/38, 42%). Smartphone ownership was roughly split, with 45% (17/38) owning smartphones, but nearly 92% (35/38) had access to a smartphone via family members. See Table 1 for participant demographics.

**Table 1.** Participant demographic information (N=38).

Characteristics	Values
Age (years), mean (SD)	37 (13.7)
Sex, n (%)	
Female	30 (79)
Education level, n (%)	
No education	5 (13)
Primary	11 (29)
Middle school	16 (42)
High school	4 (11)
Undergraduate	2 (5)
Marital status, n (%)	
Married	29 (76)
Unmarried	4 (11)
Widowed	4 (11)
Divorced	1 (2)
Employed, n (%)	
Yes	12 (32)
No	26 (68)
Smartphone ownership, n (%)	
Yes	17 (45)
No	21 (55)
Average length of smartphone ownership (years), mean (SD)	3.33 (1.15)
Smartphone ownership in family, n (%)	
Yes	35 (92)
No	3 (8)

### Awareness and Understanding of DP

The FGDs revealed a general lack of awareness about DP. Initially, most participants reported to have had no prior knowledge or understanding of the concept and many participants expressed surprise upon learning about the tracking capabilities associated with DP, with responses such as, “I didn’t know someone could track my phone” (caregiver), indicating a widespread unfamiliarity with the concept. Participants initially understood DP as government or police surveillance. For instance, one participant expressed, “Good, you will be able to catch criminals*”* (caregiver), “It helps in tracking lost phones and finding people in emergencies” (patient), and “knowing the location can help in emergencies” (caregiver). After further discussion, participants began to grasp the notion of DP and its implications. Several participants acknowledged an increased understanding, with comments highlighting their comprehension of both pros and cons of DP, “Now that I understand, it has both pros and cons” (patient), and “I see now how it works; it has its advantages and disadvantages” (caregiver). This shift in understanding highlighted the importance of educational interventions to enhance awareness of DP and its potential applications.

### Types of Data Residents Are Willing to Share

Residents showed a willingness to share specific types of data, particularly they talked about communication and app usage. Participants frequently mentioned the use of their phones for making calls, with one stating, “It [phone calls], you can use that data. I use the phone mainly to make calls” (caregiver), and another noting, “I talk to my children and husband on the phone. So, it can be used maybe” (caregiver). Similarly, messaging apps such as WhatsApp were commonly used, with comments such as, “We use messaging apps like WhatsApp, so you can get that data” (patient), indicating a prevalent reliance on these platforms for communication and their opinions regarding collection of those data. Participants directly agreed that they were willing to share communication data with comment such as, “You can see how much we talk” (patient) but did not want their communication content to be seen with one participant saying, “Knowing more than they need [referring to content of communication] to is bad*”* (caregiver). App usage also emerged as a significant area of data sharing, with participants highlighting their engagement with various apps for communication and entertainment. Examples included, “I use WhatsApp, Facebook, and Bkash,” and “Apps like TikTok and YouTube are frequently used in our home” (caregiver). Participants revealed that DP can offer insights into personal preferences and behaviors, as one participant noted, “They can know who likes which drama, who listens to music, who likes music. Can understand people’s tastes” (patient).

Additionally, location data were recognized for their use, especially in emergencies, with statements such as, “Tracking [location] can help us...,*”* (patient). Participants highlighted various practical benefits of location tracking, such as its convenience for navigation and travel. One participant noted, “I keep location on my phone; it is convenient to go somewhere” (caregiver), emphasizing the everyday use of this feature. Overall, participants recognized that location services could significantly aid in navigation and safety. When asked whether it was okay for their location to be used for DP, participants were mostly in agreement with comment, such as “I think it is good. Digital phenotyping is better in this case” (patient). When asked whether it would be perceived dangerous to track people’s location, participants disagreed with comments, such as “No knowing this is not a problem. It’s better for me” (patient) and “In some circumstances it is good [referring to location tracking]” (caregiver).

### Perceived Benefits and Risks

Participants identified several benefits of DP, particularly in health monitoring. Participants highlighted the potential of smartphones and apps to improve the management of mental health conditions. Participants made comment such as, “It will be very helpful for monitoring health conditions*”* (caregiver) and “Health monitoring through apps can provide immediate assistance” (caregiver). They highlighted the convenience and usefulness of receiving counseling and medical treatment through mobile phones, with one participant noting, “If you receive counselling, get treatment and diagnosis through mobile phones, it will be very helpful” (patient). The immediacy and accessibility of health monitoring through apps and opportunities were appreciated, with practical benefits in managing treatment schedules and medication reminders. Overall, the perceived benefits of DP among participants were substantial for improved health monitoring and management, highlighting the potential of digital tools to improve overall well-being.

Despite the general willingness to share data, participants expressed considerable concerns regarding privacy and data security. Many were apprehensive about the potential misuse of their personal information. One participant articulated this fear: “Someone will know your personal information; is that not a bad side of it?” (caregiver). This sentiment was widely shared, with participants expressing fears that their private conversations and activities could be monitored and exploited. A participant highlighted this anxiety, saying, "Fear, because if I go to tell any information to someone, they are recording these things behind me; if my secret is leaked, I may be in danger” (caregiver). Another added, “Some people can harm us with our personal information” (patient), underscoring the potential risks associated with data breaches. There was also a sense of resignation among some participants regarding surveillance. One participant mentioned, “No problem, we are already being watched” (patient), reflecting a belief that privacy invasions are already a part of their reality. This feeling was further echoed by another participant, “We have no problem if you track. We have no problem if you hack*”* (caregiver), indicating a degree of desensitization to privacy concerns.

Participants also expressed nuanced views about the tracking and monitoring of data. While some acknowledged the necessity of such measures for security purposes, they were wary of their potential for misuse. For instance, one participant remarked, “If they want to track a criminal they can; I didn’t do anything, why should they track mine” (patient), highlighting a concern that innocent individuals might be unjustly monitored. Another participant questioned the motives behind data collection, stating, “I don’t know why. But they take it for their own interests*”* (caregiver), suggesting skepticism toward the intentions.

The concern extended to the practical usability and accessibility of digital tools, particularly among older family members. One participant pointed out, “It can be challenging to teach older family members how to use health apps” (caregiver), emphasizing the need for user-friendly interfaces and adequate support systems. Another participant noted, “It saves time and travel costs, but not everyone knows how to use the apps properly” (patient), indicating that technological literacy remains a significant barrier.

In summary, while there is a willingness among residents to share data for the benefits they might bring, significant concerns about privacy invasion and data misuse persist. These concerns are coupled with practical challenges related to the usability of digital tools, particularly for older and less tech-savvy individuals.

### Adaptation of DP Tools

The discussions underscored the necessity for DP tools to be culturally sensitive and user-friendly to ensure broad acceptance. Participants emphasized that these tools should reflect local customs and practices, as well as provide valuable information relevant to their daily lives. For instance, one participant mentioned, “Health apps could offer valuable information and support” (caregiver), highlighting the potential benefits of culturally tailored digital health solutions and information.

Additionally, adapting data collection methods to local behaviors and technological habits is essential. Given the high use of communication apps such as WhatsApp and social media platforms such as Facebook and TikTok, DP tools could focus on analyzing data from these sources to identify behavioral patterns relevant to mental health. As a participant noted, “We use WhatsApp, Facebook, and YouTube a lot; you can see our activities there” (caregiver).

Furthermore, addressing privacy concerns through robust data protection measures and transparent communication can build trust among users. One participant expressed, “If we know our data is safe and not misused, we will feel more comfortable sharing it” (patient). Incorporating local community feedback into the design of these tools can ensure that they meet the specific needs and concerns of the population. Another participant highlighted, “If you involve us in the process, we can tell you what works best for us” (caregiver).

## Discussion

### Principal Results

By understanding the local context and involving the community in the research process, this study aims to create sustainable and impactful approach into DP research. The findings from this study highlight both the potential and the challenges associated with implementing DP in LMICs, particularly in underserved communities such as the Korail slum in Dhaka, Bangladesh. The results indicate a general lack of awareness and understanding of DP among residents, though there is significant interest and perceived benefit.

The initial unfamiliarity with DP shows the need for targeted educational interventions. Despite the widespread use of smartphones in the community, most participants had not previously encountered the term or understood the concept. This lack of awareness could hinder the adoption of DP tools, as individuals are unlikely to engage with technologies they do not understand. Educational initiatives should therefore focus on explaining how DP works and its potential benefits in health [1].

Once informed about DP, participants recognized several significant benefits, particularly in the realm of health monitoring. The convenience and use of location tracking in emergencies were noted as important advantages. Participants saw potential in using smartphones and apps to manage mental health conditions. This reflects a broader trend in digital health where mobile health apps are increasingly recognized for their ability to provide immediate assistance, manage treatment schedules, and offer valuable health-related information [10].

One critical barrier to implementing DP in the context of LMICs such as Korail is the low rate of individual smartphone ownership. In this study, only 45% (17/38) of the participants reported owning a smartphone, with many (35/38, 92%) relying on shared devices within their families. This dynamic presents a significant challenge to the feasibility of DP, which typically requires continuous, personal data collection via smartphones.

In many LMICs, it is not uncommon for families to share a single smartphone, which raises questions about the reliability of the data collected [24]. A shared device could distort the individual data necessary for accurate mental health monitoring, especially if different family members use the same phone throughout the day. While participants did not explicitly raise this as a concern in the focus groups, it is essential to consider whether shared use of a “family smartphone” is culturally normalized to the point that participants do not view it as problematic.

To address this challenge, future iterations of DP interventions in LMICs could explore the development of family-centered monitoring systems. Such systems could account for shared device use by developing mechanisms that differentiate between users or adapt to intermittent access by the primary individual being monitored. For example, the technology could include features that identify individual users at the start of each “session” (such as logging the user upon unlocking the phone with their fingerprint) or leverage novel machine learning algorithms that account for shared usage patterns.

The results of this study demonstrate a clear lack of awareness and understanding of DP among the residents of Dhaka’s Korail slum. Most participants had not encountered the concept prior to the FGDs, revealing a significant knowledge gap. While participants expressed willingness to share certain types of data, such as communication and app usage, they were apprehensive about more intrusive forms of data collection, particularly if it involved accessing the content of their communications. This mirrors concerns raised in other LMIC contexts, where distrust in data handling and fear of surveillance are common [24,25]. These concerns must be addressed through transparent communication about how data will be used and protected, as well as the implementation of robust data security measures. Failure to address these issues could significantly impede the adoption of DP tools in slum communities. In many LMICs, the legal infrastructure necessary to safeguard personal data is either underdeveloped or inconsistently enforced, leaving individuals vulnerable to potential misuse of their information. This distrust is compounded by participants’ feelings of resignation regarding surveillance, as many believe that they are already being monitored in some form. This perception represents a substantial hurdle to overcome in implementing any digital health tool. Moreover, the digital divide—characterized by uneven access to technology and varying levels of technological literacy—remains a critical issue. Although smartphone ownership in the Korail slum is relatively high, a significant proportion of residents still rely on family members’ devices.

### Comparison With Available Literature and Implications

Despite the perceived benefits, concerns about privacy and data security were prominent. Participants expressed fears that their personal information could be misused, with some feeling resigned to the idea that they are already being watched. This highlights a critical barrier to the adoption of DP tools: the need to ensure robust data protection measures and communicate these effectively to users. Participants in HICs have similar concerns to those in LMICs regarding data sharing, particularly in terms of privacy, data security, and ethical considerations. In HICs, participants express significant concerns about privacy and the potential misuse of their personal data. These concerns include the inadequacy of current regulations to protect sensitive personal information and the potential for data to be sold or analyzed outside the health care system [26].

Transparency, consent, accountability, and fairness are also critical issues. There is a strong emphasis on ensuring that DP tools are developed with robust data protection measures and clear communication about these measures to build trust [27,28]. In both HICs and LMICs, the willingness to share data is often contingent upon understanding how the data will be used and ensuring that robust measures are in place to protect privacy. Addressing these concerns through transparent practices and community engagement is crucial for the acceptance and effectiveness of DP technologies. While privacy is a universal issue, the extent and nature of these concerns can vary significantly. Participants from HICs generally have greater awareness and more robust expectations of privacy protection mechanisms, often influenced by stringent data protection regulations and a higher level of digital literacy [28]. In contrast, residents of Korail, despite recognizing the importance of privacy, might exhibit a certain resignation toward surveillance, possibly due to their frequent exposure to informal and less regulated data environments. Building trust through transparent data practices and addressing privacy concerns directly will be essential to foster acceptance [16,29].

The necessity for DP tools to be culturally sensitive and user-friendly was also emphasized. Participants stressed that these tools should reflect local customs and practices and be accessible to all, including older and less tech-savvy individuals. This points to the importance of designing digital health tools that are not only technologically effective but also culturally relevant and easy to use. Engaging the community in the design and implementation process can enhance the relevance and acceptance of these tools [30].

The findings suggest several practical implications for the implementation of DP in LMICs. There is a need for comprehensive educational initiatives to raise awareness and understanding of DP. These programs should use local languages and culturally relevant metaphors to explain the technology and its benefits [14]. Robust measures must be in place to protect users’ data, and clear communication about these measures can help alleviate fears and build trust among users [17]. DP tools should be adapted to fit the cultural context of the target population, possibly involving modifications to the types of data collected or the way data are interpreted to ensure that they are relevant and acceptable [31]. The usability of digital tools is crucial, particularly for older and less technologically literate individuals. Tools should be designed with these users in mind, providing simple interfaces and adequate support systems [32]. Involving the community in the design, testing, and deployment of DP tools can improve their acceptability and effectiveness. Participatory approaches ensure that interventions are tailored to meet the specific needs and preferences of the community [33].

### Strengths and Limitations

Despite the valuable insights gained from this study, several limitations should be acknowledged. First, the sample size of 38 participants, although sufficient for qualitative analysis, may not fully capture the diverse experiences and perspectives of all residents within the Korail slum. This limitation in sample diversity could affect the generalizability of the findings to other slum communities or urban poor populations in different LMIC settings [34]. Second, the reliance on FGDs as the primary data collection method may have influenced participants’ responses due to social desirability bias. Participants might have felt pressured to conform to perceived group norms or expectations, potentially limiting the expression of dissenting views or concerns about DP [35]. Another limitation is the potential for translation. DP is an emerging technology [33], and it might be difficult to understand in general, and in the FGD the meaning of DP was explained in Bengali from an English explanation. This process might have introduced nuances or misinterpretations among participants [36]. Ensuring accurate and culturally sensitive translation is critical, but even with meticulous efforts, some meaning can be lost or altered. Moreover, the study’s design captures a snapshot of participants’ perceptions and attitudes at a single point in time. Longitudinal research could provide a more comprehensive understanding of how awareness, acceptance, and concerns about DP evolve over time, particularly as participants become more familiar with the technology [37]. Technological limitations also played a role. Although smartphone usage is relatively common, the varying degrees of technological literacy among participants could influence their understanding and engagement with DP tools. Future studies should consider incorporating training sessions to ensure that all participants have a baseline understanding of the technology being discussed [38]. Finally, while this study focused on a specific slum in Dhaka, the findings might not be directly applicable to other urban or rural settings in Bangladesh or other LMICs without considering local cultural, social, and economic contexts. Comparative studies across different settings are necessary to validate and expand upon these findings [39].

Finally, integrating DP tools into existing health systems can be challenging. Health systems in LMICs are often underresourced and fragmented, which can complicate the implementation of new technologies. Successful integration requires collaboration with local health authorities and stakeholders to align DP initiatives with national health priorities and infrastructure. Training health care providers to use and interpret DP data is also crucial to ensure that the technology can be effectively used in clinical practice.

### Conclusions

This study shows the potential of use of DP to improve mental health care in underserved communities such as the Korail slum. However, for these tools to be effective, it is crucial to address the existing barriers related to awareness, privacy, cultural sensitivity, and usability. By focusing on educational initiatives, robust data protection, cultural adaptation, user-friendly design, and community engagement, DP can become a significant tool in bridging the mental health care gap in LMICs. Further research is needed to develop and implement these solutions in a way that is both effective and sustainable.

## Supplementary material

10.2196/65530 Multimedia Appendix 1 Coding tree illustrating key themes and subthemes derived from the thematic analysis of focus group discussions on digital phenotyping awareness and acceptance among individuals with serious mental disorders and their caregivers in the Korail slum, Dhaka, Bangladesh.

## Abbreviations:

COREQ: Consolidated Criteria for Reporting Qualitative Research
DP: digital phenotyping
FGD: focus group discussion
HIC: high-income country
LMIC: low- and middle-income country
SMD: serious mental disorder
