# Supplementary material for: Investigating Awareness and Acceptance of Digital Phenotyping in Dhaka’s Korail Slum: Qualitative Study
Source: JMIR Form Res. 2025 Jun 23;9:e65530. doi: 10.2196/65530 (PMC12207998; doi:10.2196/65530)
Supplement: Multimedia Appendix 1 [file formative-v9-e65530-s001.docx]

**Topic Guide**

**Welcome**

- Welcome all participants.
- Introduce myself, research assistant and the project aim.
- Ice breaker activity for members of the FGD (e.g., please say your name and your favorite food).
- Explain housekeeping rules – e.g., be polite to each other, don’t talk over each other, keep everything discussed in the FGD private, this is a safe space to discuss your opinions – there are no right or wrong answers.
- Explain that the FGD is going to be recorded.
- Ask if anybody has any questions.

**Background**

- Ask participants to review Participant Information Sheet Easy Read so everybody has a brief understanding of what digital phenotyping is.
- Ask participants to complete the demographic questionnaire (this will take 5-10 minutes)
- [Start Audio Recording]
  - State date, time and type of discussion being held.
  - Ask participants to say their name again for the purpose of being able to assign an ID number to the participants voice when transcribing the FGD.

**Mobile Phone Use**

1. What type of phone do you use?

If smartphone, what type of smartphone do you use?

1. Besides calling and messaging, what do you use your smart phone for?
2. How many members of your family use smartphones?
3. Besides calling and messaging, what do they use their smart phone for?
4. What type of phones do your neighbors use? How many prefer smartphones?
5. How many people in Korail do you think use smartphones?
6. In general, besides calling and messaging, what do you think most people use their smartphones for?

**Familiarity to Online Mental Health/Data Collection Tools**

1. Do you know of any online mental health service that can be provided through smartphones? If so, what was it/were they?
2. What is your opinion regarding them? Can they be improved?
3. What is your opinion regarding using your phone for detection and monitoring of health problems?

**Digital Phenotyping**

*The concept of digital phenotyping will be explained to the participants before the following questions as a reminder.*

*Digital phenotyping is a method through which data is collected from smartphones and other smart devices to understand individual’s behavior from their regular daily activities. Researchers can use this data to study social cohesion and interactions, behavioral patterns, speech, mobility etc., which are known as digital phenotypes.*

*Data from the phone includes phone usage, location, and use of social media (such as Facebook, TikTok). An application is installed on participants’ phones after explaining what sort of data will be collected from them, which will allow researchers to collect that data. The data will only be accessible to those involved in the study. The data generated by the app can be used to identify potential mental health problems which can be understood through phone usage patterns.*

1. Do you understand what digital phenotyping is?
2. What do you think of digital phenotyping?
3. Do you think it can be a useful tool?
4. What particular aspects of digital phenotypes do you think is acceptable – for instance, location, social media, screen behavior, call logs etc.
5. Will you be comfortable sharing your data this way?
6. Do you think people in this community will be comfortable sharing your data this way?
7. Do you have any concerns with digital phenotyping? If so, what are they?
8. Do you think people with mental health issues will be comfortable with digital phenotyping?
9. What problems might arise?
